# Risk Factors for Ischemic Stroke in Atrial Fibrillation Patients Undergoing Radiofrequency Catheter Ablation

**DOI:** 10.1038/s41598-019-43566-z

**Published:** 2019-05-07

**Authors:** Yun Gi Kim, Jaemin Shim, Suk-Kyu Oh, Kwang-No Lee, Jong-Il Choi, Young-Hoon Kim

**Affiliations:** Arrhythmia Center, Korea University Medical Center Anam Hospital, Seoul, Republic of Korea

**Keywords:** Interventional cardiology, Outcomes research

## Abstract

Ischemic stroke after radiofrequency catheter ablation (RFCA) in atrial fibrillation (AF) patients is a great challenge for electrophysiologists. We performed this retrospective study to evaluate clinical and echocardiographic characteristics associated with increased risk of ischemic stroke following RFCA. A total of 2,352 consecutive patients with AF who underwent first-time RFCA were analyzed. Among 10,023 patient*year follow up, ischemic stroke occurred in 49 patients (0.49% per year). Late recurrence after last RFCA was significantly associated with ischemic stroke (3.8% vs. 12.9%, p < 0.001). Old age (≥60 years old) (3.2% vs. 15.4%, p = 0.001), non-paroxysmal AF (hazard ratio = 1.91, p = 0.024), left atrium (LA) size ≥45.0 mm (6.6% vs. 11.7%, p < 0.001), E over E’ ≥10 (4.3% vs. 20.1%, p < 0.001), dense spontaneous echo contrast (SEC) (5.2% vs. 19.0%, p = 0.006), and decreased left atrial appendage (LAA) flow velocity (≤40 cm/sec) (4.1% vs. 10.8%, p < 0.001) were also associated with increased risk of ischemic stroke. The REVEEAL score derived from the risk factors identified in this study was superior to CHA_2_DS_2_-VASc score (p < 0.001) for the prediction of ischemic stroke. In conclusion, the risk factors for ischemic stroke in post-RFCA AF patients are not identical to RFCA naive AF patients and different approach to stratify the risk of ischemic stroke is needed.

## Introduction

Atrial fibrillation (AF) is a major risk factor for ischemic stroke, and depending on CHA_2_DS_2_-VASc score, the annual incidence of ischemic stroke in AF patients ranges between 0.2% and 12.2%^1^. Therefore, the prevention of ischemic stroke with anticoagulants is the cornerstone in managing AF patients^1–3^.

Radiofrequency catheter ablation (RFCA) is now considered a treatment of choice in highly symptomatic AF patients who are refractory to antiarrhythmic drug (AAD). As compared to AAD, RFCA is associated with significantly higher rate of any atrial tachycardia free survival and better quality of life^4,5^. Recent randomized controlled trials also suggest that RFCA might be superior to AAD as a first-line treatment strategy^6^. Although there is no randomized clinical trial demonstrating the superiority of RFCA compared to medical treatment in terms of ischemic stroke, recent propensity score matched retrospective analysis using Swedish Patient Register suggested that RFCA is associated with significant reduction in future risk of ischemic stroke as compared with medical therapy alone^7^. The beneficial effect of RFCA was driven primarily by patients with CHA_2_DS_2_-VASc ≥2^7^. Another retrospective analysis performed with Danish administrative registries revealed that maintenance of oral anticoagulation beyond 3 months after RFCA was associated with significantly increased risk of serious bleeding without reducing the risk of ischemic stroke^8^. Maintenance of oral anticoagulation did not reduce ischemic stroke even in patients with CHA_2_DS_2_-VASc ≥2 which suggests that CHA_2_DS_2_-VASc score is not sufficient for thromboembolic risk stratification in post-RFCA AF patients^8,9^. The annualized incidence (per 100 patient*year follow up) was 0.70% in Swedish registry and 0.55% in Danish registry which was relatively low considering mean CHA_2_DS_2_-VASc scores of both registry^7,8^. Therefore, routine anticoagulation may not be appropriate in post-RFCA AF patients because previous study suggests annual incidence of ischemic stroke over 1.7% and 0.9% is needed to justify the use of warfarin and non-vitamin K oral antagonist (NOAC), respectively^10^. Furthermore, it is possible that the risk factors for ischemic stroke differ between RFCA naive AF patients and post-RFCA AF patients. Therefore, we aimed to identify clinical risk factors associated with ischemic stroke in patients undergoing RFCA and to develop a new scoring system suitable for identifying post-RFCA AF patients with true high risk of suffering future ischemic stroke for whom anticoagulation is justified.

## Methods

The aim of this study was to evaluate clinical and echocardiographic characteristics associated with future risk of ischemic stroke in AF patients undergoing RFCA. After identifying individual risk factors, we created integrated scoring system based on these risk factors to predict the future risk of ischemic stroke. We also compared the efficacy of this scoring system with conventional CHA_2_DS_2_-VASc scoring system.

### Patients

All AF patients undergoing first-time RFCA in Korea University Anam Hospital between June 1998 and May 2016 were retrospectively analyzed. There was no specific exclusion criteria. The protocol of the current study was consistent with the ethical guidelines of the 2008 Helsinki Declaration. Institutional Review Board of Korea University Anam Hospital ensured appropriate ethical and bioethical conduct and approved this study. Written informed consent was waived due to its retrospective nature. All patient records and medical information were anonymized prior to analysis.

### Radiofrequency catheter ablation

Protocols of RFCA in our institution are published elsewhere^3,11–13^. Briefly, multiple introducer sheaths were positioned into femoral vessels by Seldinger’s method. A quadripolar, decapolar, and duo-decapolar catheters were positioned at right ventricle or superior vena cava, high right atrium, and coronary sinus, respectively. Continuous blood pressure monitoring and blood sampling were conducted by left femoral artery line. Double trans-septal punctures were performed using Brockenbrough needle, and two SL1 sheaths. Just before trans-septal puncture, intravenous unfractionated heparin was injected and maintained with target activated coagulation time of 300–350 seconds. Angiographies of left atrium (LA) were performed with pig-tail catheter and automatic power injector in right oblique and left oblique view. After circular mapping and ablation catheters were positioned, LA geometry was performed. For 3 dimensional mapping system, either EnSite NavX/Velocity (St. Jude Medical, St. Paul, Minnesota) or CARTO (Biosense Webster, Irvine, California) system were used. For paroxysmal AF, 4 pulmonary vein isolation was performed and if there was non-pulmonary vein trigger, additional ablation was performed to isolate the trigger focus. Pulmonary vein isolation and additional complex fractionated atrial electrogram guided ablation or linear ablation were performed for non-paroxysmal AF based on the operator’s decision.

### Echocardiography

Before RFCA, trans-thoracic echocardiography (TTE) and trans-esophageal echocardiography (TEE) were performed. Focused evaluation of LA and left atrial appendage (LAA) was performed to reveal any evidence of spontaneous echo contrast (SEC) or thrombus. SEC was graded as very mild (minimal echogenicity, only detectable transiently, or increasing gain setting required for the detection), mild (detectable without increasing gain setting), moderate (dense, swirling echogenic material, echogenic signal is dense in LAA compared to LA), or severe (dense, swirling echogenic material, echogenic signal is equivocal in LAA and LA). Dense SEC was defined as a composite of moderate and severe SEC. Velocities of emptying (forward) and filling (backward) LAA flow were also measured.

### Definitions

In the current study, ischemic stroke was defined as any neurological symptoms lasting more than 24 hours which cannot be explained by other medical conditions. Transient ischemic attack was defined as any neurologic symptoms that were resolved completely within 24 hours which are not attributable to any other medical causes. If acute infarction lesion was observed in brain computed tomography or magnetic resonance imaging, the event was classified as ischemic stroke despite complete restoration of neurologic symptoms. Hemorrhagic stroke was defined as any neurologic symptoms accompanied by hemorrhagic lesions in brain computed tomography or magnetic resonance imaging. All patients who experienced stroke were evaluated by neurologist or neurosurgeon and future treatment strategy was determined based on collaboration among cardiologist, neurologist, and neurosurgeon. Late recurrence was defined as any atrial tachyarrhythmia lasting for more than 30 seconds occurring after 3 months of blanking period after RFCA.

### Anticoagulation

In the current study, prescription of anticoagulants was recorded in every patient throughout the total follow up period. Anticoagulants consisted of warfarin, dabigatran, rivaroxaban, apixaban, and edoxaban. We calculated anticoagulated follow up duration and non-anticoagulated follow up duration for each patient. Therefore, we were able to obtain anticoagulation coverage, which is defined as (anticoagulated duration/total follow up duration)*100%, for each individual patient and for any specific group.

### Statistics

Continuous variables are described as means ± standard deviations. Categorical variables are presented as percentile values. Continuous variables were compared with Student t test. Categorical variables were compared with chi-square test or Fisher’s exact test as appropriate. Cumulative incidence of stroke (time to first event) was depicted by Kaplan-Meier survival curve analysis and difference between groups were compared using log-rank test. Cox regression analysis was performed to calculate univariate hazard ratio (HR) and HR adjusted for individual components of CHA_2_DS_2_-VASc score. Receiver operating characteristic (ROC) curve analysis with calculation of area under curve (AUC) was performed to evaluate efficacy of CHA_2_DS_2_-VASc score and new scoring systems developed in this study to predict future risk of ischemic stroke in AF patients undergoing first-time RFCA. Comparison of two ROC curves was performed by statistical method suggested by Hanley and McNeil^14^. Missing data were excluded from each analysis and no imputation was performed. All significance tests were two-tailed and p value ≤ 0.05 were considered statistically significant. All statistical analyses were performed with SPSS version 21.0 (IBM, Armonk, NY, USA).

## Results

### Patients

Between June 1998 and June 2016, a total of 2,352 patients underwent first-time RFCA for AF. Total number of procedure was 2,997 with 546 re-do procedures and 83 tri-do procedures. A total of 2,293 (97.5%) and 2,135 (90.8%) patients underwent TTE and TEE evaluation, respectively. Baseline characteristics of the study population are summarized in Supplementary Table S1. Briefly, mean age was 55.4 ± 10.9 and 79.6% of patients were male. AF was non-paroxysmal in 40.2% of patients and mean LA size was 41.1 mm. Mean CHA_2_DS_2_-VASc score was 1.3 ± 1.3. Total follow up duration was 10,023 patient*years and 1 year follow-up rate was 83.6%. During follow up, a total of 82 all strokes occurred with annualized incidence of 0.82%. Ischemic stroke occurred in 49 patients with annualized incidence of 0.49% and transient ischemic attack in 13 patients (Supplementary Fig. S1). The location and severity of ischemic stroke in 49 patients are summarized in Supplementary Table S2.

### Risk factors for ischemic stroke

Baseline clinical and echocardiographic characteristics were compared between patients with and without ischemic stroke after RFCA (Table 1). Patients who experienced ischemic stroke after RFCA had significantly older age, larger LA, higher mean CHA_2_DS_2_-VASc score, unfavorable diastolic function profile, decreased LAA flow velocity, and higher prevalence of non-paroxysmal AF and SEC as compared with patients who did not suffer ischemic stroke.

**Table 1:** Baseline characteristics of patients with and without ischemic stroke after RFCA.

	No ischemic stroke (n = 2,303)	Ischemic stroke (n = 49)	p value
Age (year)	55.2 ± 10.9	62.0 ± 10.1	0.000
Male sex	1,836 (79.7%)	36 (73.5%)	0.283
Body weight (kg)	70.8 ± 11.1	66.9 ± 10.9	0.017
Height (cm)	168.2 ± 8.2	164.9 ± 9.3	0.007
BMI	25.0 ± 3.0	24.5 ± 3.1	0.336
Non-paroxysmal	918 (39.9%)	27 (55.1%)	0.031
AF duration (year)	4.7 ± 4.7	5.6 ± 4.7	0.187
Heart failure	174 (7.6%)	6 (12.2%)	0.268
Hypertension	849 (36.9%)	16 (32.7%)	0.545
Diabetes mellitus	254 (11.0%)	5 (10.2%)	0.855
Previous CVA, TIA, or embolism	179 (7.8%)	6 (12.2%)	0.275
Vascular disease	213 (9.2%)	7 (14.3%)	0.216
CHA_2_DS_2_-VASc	1.2 ± 1.3	1.7 ± 1.8	0.105
TTE findings			
LA size (mm)	41.1 ± 6.0	43.8 ± 4.8	0.002
LV ejection fraction	54.9 ± 6.2	55.3 ± 6.1	0.654
E	65.7 ± 16.9	76.7 ± 19.8	0.000
E’	8.0 ± 2.4	7.5 ± 2.2	0.272
E over E’	8.9 ± 4.0	11.1 ± 3.9	0.003
TEE findings			
LAA emptying velocity (cm/sec)	47.9 ± 21.8	33.1 ± 19.0	0.000
LAA filling velocity (cm/sec)	49.7 ± 22.3	37.8 ± 20.3	0.001
LAA average velocity (cm/sec)	48.8 ± 20.9	35.4 ± 18.8	0.000
SEC	442 (21.1%)	14 (36.8%)	0.019
Dense SEC	72 (3.4%)	5 (13.5%)	0.010
Thrombus	4 (0.2%)	1 (2.6%)	0.086
Hemoglobin (g/dl)	14.7 ± 1.4	14.2 ± 1.3	0.036
WBC (10^3^/μl)	6.5 ± 3.4	6.6 ± 1.7	0.769
Platelets (10^3^/μl)	207.6 ± 49.2	205.9 ± 52.0	0.806
Creatinine (mg/dl)	1.0 ± 0.4	1.0 ± 0.2	0.999

AF: atrial fibrillation; BMI: body mass index; CVA: cerebrovascular accident; LA: left atrium; LAA left atrial appendage; LV: left ventricle; RFCA: radiofrequency catheter ablation; SEC: spontaneous echo contrast; TEE: transesophageal echocardiography; TIA: transient ischemic attack; TTE: transthoracic echocardiography; WBC: white blood cell.

**Figure 1:**
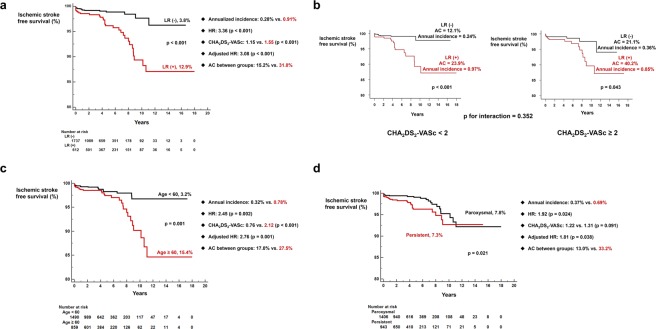
Influence of clinical parameters on ischemic stroke. (**a**) Kaplan-Meier curve analysis of cumulative incidence of ischemic stroke according to the presence late recurrence after last RFCA. Adjusted HR was calculated by adjusting individual component of CHA_2_DS_2_-VASc score. (**b**) Clinical impact of late recurrence after last RFCA in patients with CHA_2_DS_2_-VASc <2 and ≥2. (**c**,**d**) Patients with age ≥60 and non-paroxysmal AF had significantly increased risk of ischemic stroke despite higher anticoagulation coverage. AC: anticoagulation coverage; HR: hazard ratio; LR: late recurrence; RFCA: radiofrequency catheter ablation.

Patient who experienced late recurrence after last RFCA showed significantly higher cumulative incidence (3.8% vs. 12.9%, log-rank p < 0.001; Fig. 1a) and annualized incidence (0.28% vs. 0.91%) of ischemic stroke as compared with patients who maintained sinus rhythm throughout the whole follow up period despite higher anticoagulation coverage (15.2% vs. 31.8%). HR adjusted for individual components of CHA_2_DS_2_-VASc score was 3.08 (p < 0.001). Late recurrence after last RFCA was associated with higher risk of future ischemic stroke irrespective of whether CHA_2_DS_2_-VASc score was <2 or ≥2 (p for interaction = 0.352; Fig. 1b). Among 1,529 patients with CHA_2_DS_2_-VASc score <2, 27 patients experienced ischemic stroke after RFCA. Clinical and echocardiographic characteristics of these 27 patients who suffered ischemic stroke despite low CHA_2_DS_2_-VASc score are summarized in Supplementary Table S3. Patients with CHA_2_DS_2_-VASc <2 who experienced late recurrence showed significantly higher risk of ischemic stroke compared to patients with CHA_2_DS_2_-VASc ≥2 without late recurrence (5.9% vs. 12.9%, log-rank p = 0.011; Supplementary Fig. S2). Patients over 60 years old experienced significantly higher risk of ischemic stroke (3.2% vs. 15.4%, log-rank p = 0.001; annualized incidence = 0.32% vs. 0.78%; adjusted HR = 2.76, p = 0.001; Fig. 1c) despite higher anticoagulation coverage as compared to patients under 60 years old (17.0% vs. 27.5%). Non-paroxysmal AF (HR = 1.91, p = 0.024; annual incidence = 0.37% vs. 0.69%; adjusted HR = 1.81, p = 0.038; Fig. 1d) was also associated with increased risk of ischemic stroke despite higher anticoagulation coverage (13.0% vs. 33.2%).

**Figure 2:**
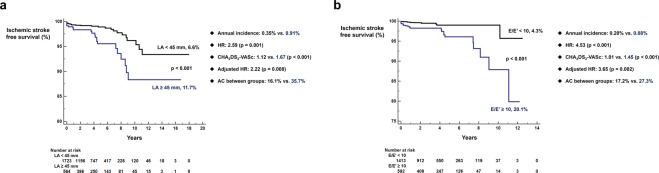
TTE risk factors for ischemic stroke. LA size ≥45.0 mm (**a**) and E over E’ ≥10 (**b**) were associated with increased risk of ischemic stroke. AC: anticoagulation coverage; HR: hazard ratio; LA: left atrium; TTE: trans-thoracic echocardiography.

**Figure 3:**
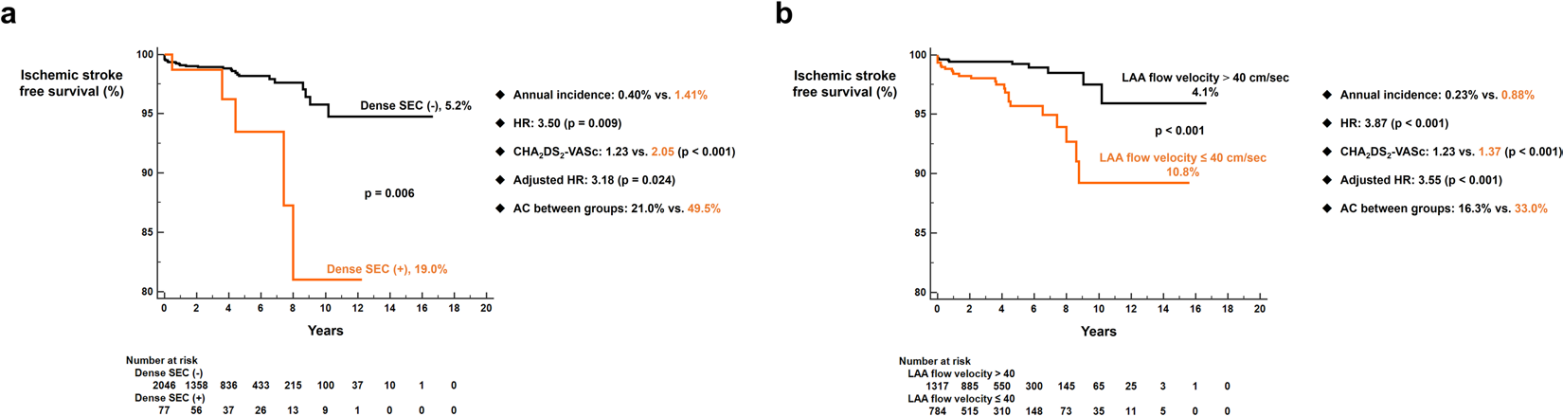
TEE risk factors for ischemic stroke. Presence of dense SEC (**a**) and decreased LAA flow velocity (**b**) were associated with increased risk of ischemic stroke. AC: anticoagulation coverage; HR: hazard ratio; LAA: left atrial appendage; SEC: spontaneous echo contrast; TEE: trans-esophageal echocardiography.

TTE findings were substantially important to predict future risk of ischemic stroke. Patients with LA size over 45.0 mm had a significantly increased risk of ischemic stroke following RFCA (6.6% vs. 11.7%; log-rank p < 0.001; annualized incidence = 0.35% vs. 0.91%; adjusted HR = 2.22, p = 0.008; Fig. 2a). E over E’ ≥10 was also associated with substantially higher risk of ischemic stroke (4.3% vs. 20.1%; log-rank p < 0.001; annualized incidence = 0.20% vs. 0.88%; adjusted HR = 3.65, p = 0.002; Fig. 2b). Presence of dense SEC (5.2% vs. 19.0%; log-rank p = 0.006; annualized incidence = 0.40% vs. 1.41%; adjusted HR = 3.18, p = 0.024; Fig. 3a) and decreased left atrial appendage flow velocity (≤40 cm/sec) (4.1% vs. 10.8%, log-rank p < 0.001; annualized incidence = 0.23% vs. 0.88%; adjusted HR = 3.55, p < 0.001; Fig. 3b) in TEE were also important predictors of ischemic stroke in AF patients undergoing RFCA. Anticoagulation coverage was higher in patients with aforementioned unfavorable TTE and TEE findings but these patients experienced higher risk of ischemic stroke following RFCA (Figs 2 and 3).

Our analyses revealed that aforementioned risk factors for ischemic stroke were also significant risk factors for ischemic stroke plus TIA. HR and HR adjusted for individual components of CHA_2_DS_2_-VASc score of each risk factor are summarized in Supplementary Table S4.

### REVEEAL and REAL score

**Figure 4:**
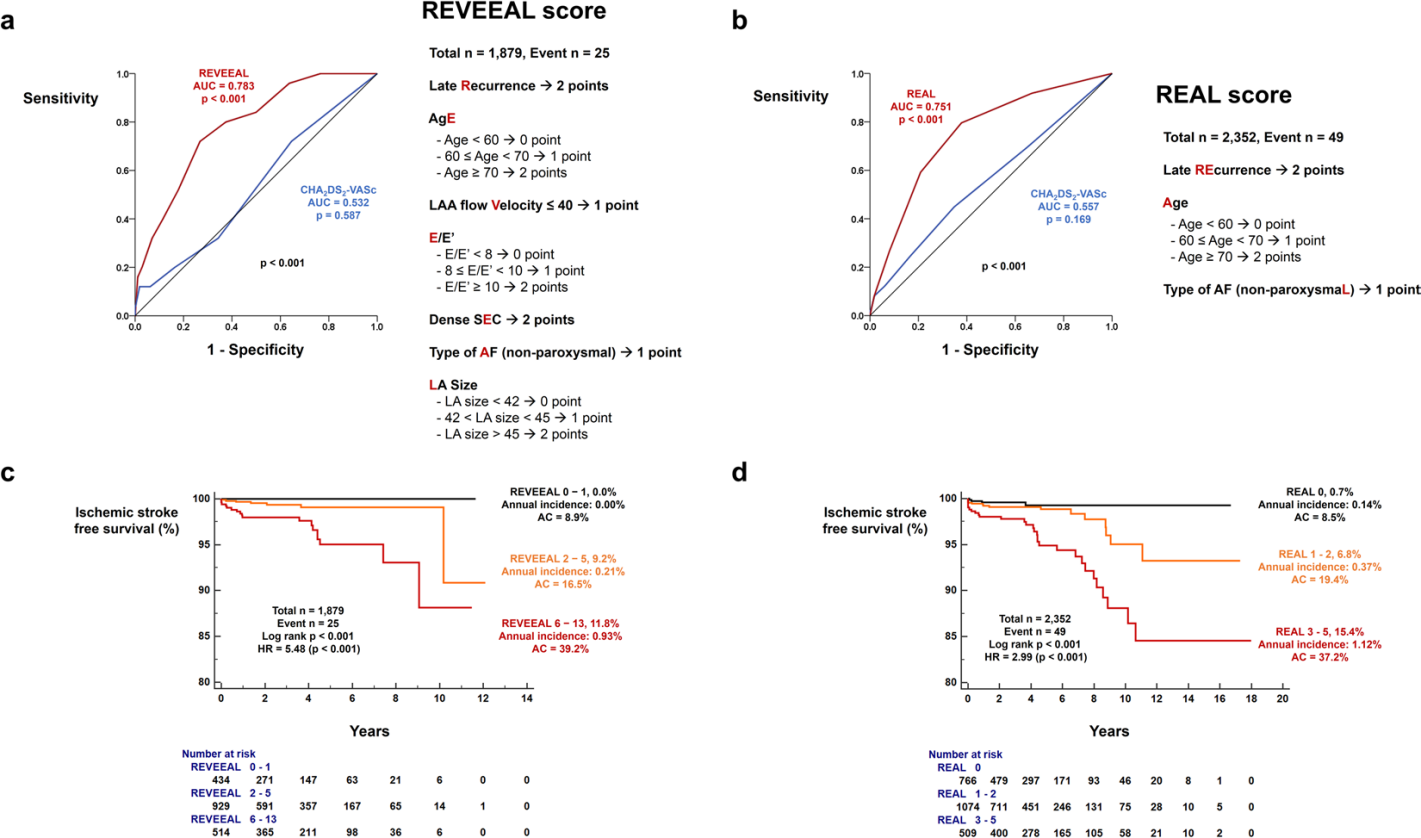
Predictive value of REVEEAL and REAL scoring system for ischemic stroke in post-RFCA AF patients. (**a**) ROC curve analysis for REVEEAL and CHA_2_DS_2_-VASc scores. (**b**) ROC curve analysis for REAL and CHA_2_DS_2_-VASc scores. (**c**) Risk of ischemic stroke according to tertile distribution of REVEEAL score. (**d**) Risk of ischemic stroke according to tertile distribution of REAL score. AF: atrial fibrillation; AUC: area under curve; HR: hazard ratio; LA: left atrium; LAA: left atrial appendage; RFCA: radiofrequency catheter ablation; ROC: receiver operating characteristic.

In the current study, CHA_2_DS_2_-VASc score was not a risk factor for post-RFCA ischemic stroke (HR = 1.22, p = 0.487; Supplementary Fig. S3). Furthermore, CHA_2_DS_2_-VASc score was not able to predict future risk of ischemic stroke in ROC curve analysis (AUC = 0.557, p = 0.169). We created a new scoring system named REVEEAL score based on the presence of late recurrence, old age (≥60 years old), type of AF, LA size, E over E’, dense SEC, and decreased LAA flow velocity (≤40 cm/sec) (Fig. 4a) and REAL score based on the presence of late recurrence, old age, and type of AF (Fig. 4b). In contrast to CHA_2_DS_2_-VASc score, REVEEAL (AUC = 0.783, p < 0.001; Fig. 4a) and REAL (AUC = 0.751, p < 0.001; Fig. 4b) scoring systems were able to predict future risk of ischemic stroke. Comparison of two ROC curves revealed that REVEEAL and REAL scoring systems were superior to CHA_2_DS_2_-VASc system (p < 0.001 for both). Significantly different risk of ischemic stroke was observed among tertiles of REVEEAL (HR = 5.48, p < 0.001; Fig. 4c) and REAL (HR = 2.99, p < 0.001; Fig. 4d) score with higher incidence of ischemic stroke in high tertile group despite higher anticoagulation coverage. Direct comparison of REVEEAL and REAL scoring systems showed statistical tendency for better prediction of ischemic stroke when TTE and TEE findings were added to clinical parameters (AUC = 0.783 vs. 0.714, p = 0.069; Supplementary Fig. S4).

## Discussion

The primary findings of the current study can be summarized as follows: (i) the incidence of ischemic stroke after RFCA was 0.49% per year with a mean CHA_2_DS_2_-VASc score of 1.3; (ii) in this registry of post-RFCA AF patients, CHA_2_DS_2_-VASc score did not predict future risk of ischemic stroke; (iii) the clinical and echocardiographic risk factors for ischemic stroke was the presence of late recurrence, old age, type of AF, LA size, E over E’, dense SEC, and decreased LAA flow velocity; (iv) in contrast to CHA_2_DS_2_-VASc score, REVEEAL and REAL scoring systems were able to predict future risk of ischemic stroke. The strong point of the current study can be summarized as follows: (i) prescription history of anticoagulants was extensively reviewed for all patients and for total follow up duration. Therefore, we were able to calculate mean anticoagulation coverage for individual patients and for specific group; (ii) all consecutive patients in our institution were included in the analysis to minimize any potential bias; (iii) TEE was performed in more than 90% of patients. Therefore, we were able to integrate not only TTE but also TEE findings to conventional clinical parameters.

### AF, RFCA, and ischemic stroke

There are 2 major theories explaining the association between AF and ischemic stroke: rhythm and substrate^15^. In atrial rhythm theory, increased risk of ischemic stroke is the result of blood stasis in LA and LAA which is caused by AF. Decreased flow velocity in LA or LAA is associated with development of SEC in which thrombus formation and embolization are likely to occur^11,16–19^. Substrate theory focuses on the inability of rhythm control therapy, although strongly challenged by recent studies, to reduce ischemic stroke and lack of temporal association between paroxysmal AF and ischemic stroke in prolonged rhythm monitoring^15,20^. It is suggested that not only blood stasis but also atrial remodeling leading to fibrotic and prothrombotic atrial cardiomyopathy plays a significant role^15^. Irrespective of underlying mechanism, anticoagulation with warfarin or NOACs is associated with significant reduction of ischemic stroke^21,22^. However, whether RFCA is associated with a reduction of ischemic stroke is under active debate. Previous studies suggested that RFCA do not reduce the risk of ischemic stroke^23^. However, recent studies report a significant reduction in the risk of ischemic stroke in AF patients undergoing RFCA^7,8,24^. Friberg *et al*. demonstrated in their propensity score matched analysis that AF patients undergoing RFCA experienced a 31% reduction in ischemic stroke and the beneficial effect of RFCA was most dominant in patients with CHA_2_DS_2_-VASc score ≥2 with a 61% risk reduction^7^. Karasoy and his colleagues also showed that RFCA is associated with a 47% risk reduction of ischemic stroke in multivariate adjusted retrospective analysis^8^. However, there is no randomized clinical trials demonstrating superiority of RFCA over medical therapy in terms of preventing ischemic stroke and further data are needed for definitive conclusion.

### Risk factors for ischemic stroke after RFCA

The incidence of ischemic stroke in AF patients undergoing RFCA merits further attention. Annualized incidence of ischemic stroke in the studies performed by Friberg *et al*. and Karasoy *et al*. was 0.70% and 0.55% respectively^7,8^. Annualized incidence of ischemic stroke in the current study, which was 0.49%, is also similar with previous studies. Collectively, incidence of ischemic stroke after RFCA is not high and therefore, anticoagulation treatment in these post-RFCA AF patients deserves in-depth discussion. In this analysis, patients without late recurrence showed 0.28% annualized incidence of ischemic stroke which is far below the threshold for both warfarin and NOAC treatment suggested by previous study^10^. Anticoagulation coverage in the group was 15.2% and considering mandatory anticoagulation duration of 2 months after RFCA, anticoagulation was rarely prescribed. Therefore, the risk of ischemic stroke in patients without late recurrence was indeed low. Furthermore, annualized incidence of ischemic stroke in patients with CHA_2_DS_2_-VASc score ≥2 but without late recurrence was significantly low as compared to patients with CHA_2_DS_2_-VASc score <2 and who experienced late recurrence (Supplementary Fig. S2). The risk of ischemic stroke in patients with CHA_2_DS_2_-VASc score ≥2 but without late recurrence should be revisited.

CHA_2_DS_2_-VASc scoring system is a well proven risk predictor of ischemic stroke in AF patients^1^. However, the efficacy of CHA_2_DS_2_-VASc score in AF patients undergoing RFCA is not well validated. Previous studies suggest that CHADS_2_ and CHA_2_DS_2_-VASc scoring systems are not ideal to guide anticoagulation treatment in post-RFCA AF patients^8,9^. CHA_2_DS_2_-VASc scoring system also did not predict the risk of ischemic stroke in our registry with AUC near 0.5. The reason for this discrepancy is not clear. Substantial number of AF patients, especially those with paroxysmal AF, remain in sinus rhythm after RFCA and despite late recurrence, RFCA is usually associated with significant reduction in overall AF burden^25,26^, and AF burden is associated with risk of ischemic stroke^27^. Because CHA_2_DS_2_-VASc score was originally developed to predict the risk of ischemic stroke in ‘AF’ patients, the substantial reduction of AF burden after RFCA might have significant influence on the predictive value of CHA_2_DS_2_-VASc score. Low number of ischemic stroke events in patients undergoing RFCA might also affect the predictive value of CHA_2_DS_2_-VASc score. REVEEAL and REAL scores, in contrast to CHA_2_DS_2_-VASc score, were able to predict future risk of ischemic stroke in the current study with AUC over 0.7. Low tertile group of REVEEAL and REAL score represented patients with significantly low risk of ischemic stroke with 0.00% and 0.14% annualized incidence for low tertile of REVEEAL and REAL respectively. Mid and higher tertile groups of each scoring system showed significantly higher risk of ischemic stroke despite substantially higher anticoagulation coverage. The predictive value of REVEEAL and REAL scoring systems in external validation cohort should be performed and risk-benefit of anticoagulation in these low risk patients needs further investigation.

### TEE findings and ischemic stroke

The association between ischemic stroke and TEE parameters such as SEC or LAA flow velocity in AF patients is documented in previous studies^16,17^. However, clinical implication of TEE findings in post-RFCA AF patients is not fully evaluated. Our data suggest that dense SEC and decreased LAA flow velocity are significantly associated with future risk of ischemic stroke in AF patients undergoing RFCA. In patients who remain in AF despite RFCA, it is likely that TEE parameters will apply as identical to AF patients without history of RFCA. Even in patients with successful restoration of sinus rhythm through RFCA, whether decreased functional status of LA and LAA, especially in non-paroxysmal AF patients, will recover after successful ablation remains uncertain. Therefore, potential risk of ischemic stroke should be kept in mind irrespective of the results of RFCA in patients with unfavorable findings in baseline TEE evaluation.

### Limitations

We acknowledge that the current study has several shortcomings. First, this was a retrospective analysis and therefore, is not free from intrinsic limitations of retrospective analysis. Second, the number of ischemic stroke was not large which is a universal problem encountered in all studies analyzing RFCA. Incidence of ischemic stroke in post RFCA patients is far below 1%, a consistent finding observed in many studies including the current data. Third, our registry was exclusively consisted of East Asian patients and therefore, care should be taken when expanding our results to other ethnic groups. Fourth, there can be a potential selection bias between those who did and did not undergo TEE evaluation. However, substantial number of patients had TEE evaluation (90.8%) and whether to perform or not perform TEE evaluation was based on patient preference and tolerance to swallow the probe rather than medical condition such as previous history of stroke or type of AF. Fifth, although the total follow-up duration was sufficient (10,023 patient*years), the duration of the follow-up was not uniform for individual patients.

## Conclusions

The risk factors for ischemic stroke in AF patients undergoing RFCA differ from RFCA naive AF patients. Presence of late recurrence, old age, non-paroxysmal AF, large LA size, high E over E’, dense SEC, and decreased flow velocity in LAA were associated with increased risk of ischemic stroke in post-RFCA AF patients. REVEEAL and REAL scores, derived from the current study, might perform better than CHA_2_DS_2_-VASc score to predict future risk of ischemic stroke in post-RFCA AF patients.

## Supplementary information

Supplementary Tables and Figures

## Data Availability

All data generated or analyzed during this study are included in this published article (and its Supplementary Information files).
