# Supplementary material for: Risk Factors for Ischemic Stroke in Atrial Fibrillation Patients Undergoing Radiofrequency Catheter Ablation
Source: Sci Rep. 2019 May 7;9:7051. doi: 10.1038/s41598-019-43566-z (PMC6504925; doi:10.1038/s41598-019-43566-z)
Supplement: Supplementary file 1 — Supplementary Tables and Figures [file 41598_2019_43566_MOESM1_ESM.pdf]

Risk Factors for Ischemic Stroke in Atrial Fibrillation Patients Undergoing Radiofrequency Catheter Ablation

Yun Gi Kim, Jaemin Shim, Suk-Kyu Oh, Kwang-No Lee, Jong-Il Choi, and Young-Hoon Kim

Arrhythmia Center, Korea University Medical Center Anam Hospital, Seoul, Republic of Korea

*** Address for correspondence**

Jaemin Shim, MD, PhD

Arrhythmia Center, Korea University Medical Center Anam Hospital

73, Inchon-ro, Seongbuk-gu, Seoul 02841, Republic of Korea

Tel: 82-2-920-5445

Fax: 82-2-927-1478

E-mail: jaemins@korea.ac.kr

Young-Hoon Kim, MD, PhD

Arrhythmia Center, Korea University Medical Center Anam Hospital

73, Inchon-ro, Seongbuk-gu, Seoul 02841, Republic of Korea

Tel: 82-2-920-5211

Fax: 82-2-923-6030

E-mail: yhkmd@korea.ac.kr

Brief title: Strategy to reduce ischemic stroke after RFCA

Word count: 3,880

Supplementary Table S1. Baseline characteristics of study patients.

	Total population (N = 2,352)
Age (year)	55.4 ± 10.9
Male sex	1,872 (79.6%)
Body weight (kg)	70.7 ± 11.2
Height (cm)	168.1 ± 8.2
BMI	24.9 ± 3.0
Non-paroxysmal	945 (40.2%)
AF duration (year)	4.8 ± 4.7
Heart failure	180 (7.7%)
Hypertension	865 (36.8%)
Diabetes mellitus	259 (11.0%)
Previous CVA, TIA, or embolism	185 (7.9%)
Vascular disease	220 (9.3%)
CHA_2DS_2-VASc	1.3 ± 1.3
TTE findings	
LA size (mm)	41.1 ± 6.0
LV ejection fraction	54.9 ± 6.2
E	65.8 ± 17.0
E’	8.0 ± 2.4
E over E’	8.9 ± 4.0
TEE findings	
LAA emptying velocity (cm/sec)	47.6 ± 21.9
LAA filling velocity (cm/sec)	49.5 ± 22.3
LAA average velocity (cm/sec)	48.6 ± 21.0
SEC	456 (21.4%)
Dense SEC	77 (3.6%)
Thrombus	5 (0.2%)
Hemoglobin (g/dl)	14.7 ± 1.4
WBC (10^3/μl)	6.5 ± 3.4
Platelets (10^3/μl)	207.6 ± 49.2
Creatinine (mg/dl)	1.0 ± 0.4

AF: atrial fibrillation; BMI: body mass index; CVA: cerebrovascular accident; LA: left atrium; LAA left atrial appendage; LV: left ventricle; SEC: spontaneous echo contrast; TEE: transesophageal echocardiography; TIA: transient ischemic attack; TTE: transthoracic echocardiography; WBC: white blood cell.

Supplementary Table S2. Location and severity of ischemic stroke events.

Patient number	Brain imaging (CT or MRI)	Lesion location	Sequelae*	Description
1	Yes	Right MCA and PCA territory	2	Left hemiparesis
2	Yes	Left insular cortex	1	Minimal sequelae
3	Yes	Multiple embolic (both thalami, Left mid brain, pons, and both cerebellar hemisphere)	2	Bed ridden status
4	Yes	Right MCA and ACA territory	2	Significant limitations in daily life and swallowing
5	Yes	Left basal ganglia and periventricular white matter	2	Right hemiparesis
6	Yes	Multiple embolic (left superior parietal cortex, parieto-temporal, temporal, basal ganglia and both cerebellar hemispheres)	2	Death
7	Yes	Not available	1	Visual field defect
8	Yes	Left MCA	2	Right hemiparesis, global aphasia, cognitive impairment
9	Yes	Multiple embolic (left presylvian cortex, right pericallosal, and left temporo-occipital lesion)	2	Death
10	No	Not available	0	No sequelae
11	Yes	Not available	2	Right hemiparesis
12	Yes	Left frontal area	0	No sequelae
13	Yes	Right occipital lobe medial portion	1	Visual field defect
14	Yes	Not available	0	No sequelae
15	Yes	Left MCA	2	Right hemiparesis
16	Yes	Multiple embolic (right frontal, posterior temporal, parietal cerebral lobes, head of caudate nucleus, posterior aspect of basal ganglia, right frontal white matter, and right cerebellar hemisphere)	1	Minimal sequelae
17	Yes	Left posterior parieto-occipital junctional watershed zone	1	Minimal sequelae
18	No	Not available	0	No sequelae
19	Yes	Left cerebellum, SCA, and PICA territory	1	Minimal sequelae
20	Yes	Left frontal area	0	No sequelae
21	Yes	Multifocal scattered acute infarction foci on both cerebral hemispheres cortical and subcortical regions and both cerebelli	0	No sequelae
22	Yes	Multiple embolic (right medial thalamus, right cerebral peduncle, left cerebral peduncle, left posterior temporo-parietal lobe, both occipital lobes, and both cerebellar hemispheres)	1	Minimal sequelae
23	Yes	Left parieto-occipital and posterior temporal lobes	2	Disabling stroke
24	Yes	Multiple embolic (both cerebral hemispheres and cerebellar hemisphere)	2	Left hemiparesis
25	Yes	Right MCA	1	Minimal sequelae
26	Yes	Left MCA	2	Right hemiparesis
27	No	Not available	0	No sequelae

28	Yes	Left MCA	2	Right hemiparesis
29	Yes	Right MCA and PICA territory	2	Left hemiparesis
30	Yes	Left parieto-occipital area	0	No sequelae
31	Yes	Left ICA borderzone	0	No sequelae
32	Yes	Not available	0	No sequelae
33	Yes	Cerebellum	1	Walking disturbance
34	Yes	Right MCA branch territory	0	No sequelae
35	Yes	Right PCA territory	1	Mortor symptoms
36	Yes	Right proximal ICA territory	2	Left hemiparesis
37	Yes	Left MCA territory	2	Right hemiparesis
38	Yes	Periventricular whitematter of left occipital horn	0	No sequelae
39	No	Not available	2	Right hemiparesis, aphasia
40	Yes	Left PCA and Right PICA territory	2	Gait disturbance, cognitive impairment
41	Yes	Both PCA territory	0	Memory defect, right visual field defect → improved
42	Yes	Left lower paramedian pons	0	No sequelae
43	Yes	Multiple embolic (both fronto-parietal cortical or subcortical white matters and suspicious right cerebellar hemisphere)	0	Dysarthria → no sequelae
44	No	Not available	0	No sequelae
45	Yes	Left MCA territory	0	No sequelae
46	Yes	Right MCA territory	1	Gait disturbance
47	Yes	Bilateral occipital lobe	1	Visual field defect
48	Yes	Left cerebellum	0	No sequelae
49	Yes	Left frontal focal lesion	0	No sequelae

* Degree of sequelae

- 0: No sequelae. Patient is fully capable of maintaining normal daily activities.
- 1: Mimimal sequelae. Patient is capable of maintaining normal daily activities with some limitation.
- 2: Significant sequelae. Patient have significant limitation in maintaining normal daily activities.

ACA: anterior cerebral artery; ICA: internal carotid artery; MCA: middle cerebral artery; PCA: posterior cerebral artery; PICA: posterior inferior cerebellar artery; SCA: superior cerebellar artery.

Supplementary Table S3. Baseline characteristics of CHA_2DS_2-VASc < 2 patients with and without ischemic stroke.

	No ischemic stroke (n = 1,502)	ischemic stroke (n = 27)	p value
Age	51.1 ± 9.7	58.3 ± 9.3	0.000
LA size	40.2 ± 5.9	44.6 ± 4.8	0.000
LV ejection fraction	55.3 ± 5.3	55.6 ± 4.6	0.820
E	64.9 ± 16.4	78.3 ± 25.1	0.001
E'	8.6 ± 2.4	8.3 ± 2.0	0.583
E over E'	8.1 ± 3.8	10.0 ± 4.2	0.041
LAA emptying velocity	49.9 ± 21.7	31.2 ± 16.8	0.000
LAA filling velocity	51.8 ± 22.1	36.2 ± 20.8	0.001
LAA average velocity	50.9 ± 20.7	33.7 ± 17.9	0.000
Non-paroxysmal	579 (38.5%)	18 (66.7%)	0.003
Dense SEC	28 (2.1%)	2 (8.7%)	0.087

LA: left atrium; LAA: left atrial appendage; LV: left ventricle; SEC: spontaneous echo contrast.

Supplementary Table S4. HRs and adjusted HRs of risk factors for ischemic stroke or TIA.

	HR	p value	Adjusted HR	p value
Late recurrence	3.44	< 0.001	3.08	< 0.001
Age ≥ 60	2.68	< 0.001	2.73	< 0.001
Non-paroxysmal AF	2.17	0.003	2.10	0.005
LA size ≥ 45.0 mm	2.40	0.001	2.04	0.008
E over E' ≥ 10	4.05	< 0.001	3.08	0.002
Dense SEC	3.18	0.008	2.56	0.043
LAA flow velocity ≤ 40 cm/sec	3.37	< 0.001	3.07	< 0.001
CHA_2DS_2-VASc	1.27	0.005	NA	NA

AF: atrial fibrillation; HR: hazard ratio; LA: left atrium; LAA: left atrial appendage; NA: not applicable; SEC: spontaneous echo contrast; TIA: transient ischemic attack.

Online Figure Legends

Figure S1

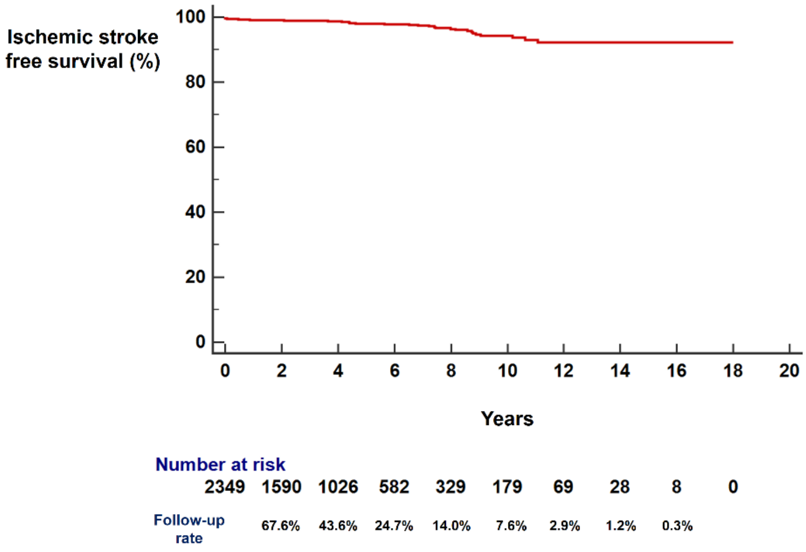

Supplementary Figure S1. The overall ischemic stroke-free survival of the whole cohort.

Patient number at risk and follow-up rate is also described.

Figure S2

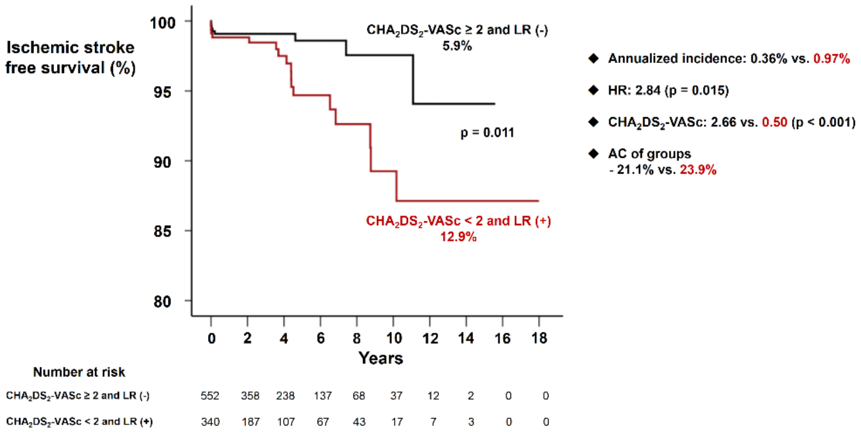

Supplementary Figure S2. Influence of late recurrence on ischemic stroke.

The risk of ischemic stroke in patients with CHA$_2$DS$_2$-VASc < 2 with late recurrence was significantly higher compared to patients with CHA$_2$DS$_2$-VASc ≥ 2 without late recurrence.

AC: anticoagulation coverage; HR: hazard ratio.

Figure S3

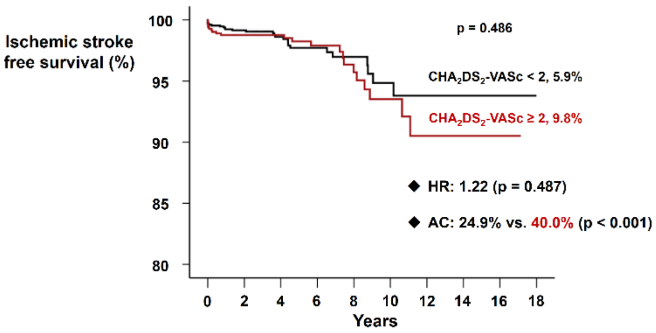

Number at risk	0	2	4	6	8	10	12	14	16	18
CHA_2DS_2-VASc ≥ 2	823	587	418	261	152	81	31	11	2	0
CHA_2DS_2-VASc < 2	1,529	1,003	608	321	177	98	38	17	6	0

Supplementary Figure S3. Influence of CHA_2DS_2-VASc score on ischemic stroke.

The cumulative incidence of ischemic stroke did not differ between patients with CHA_2DS_2-VASc < 2 and ≥ 2.

AC: anticoagulation coverage; HR: hazard ratio.

Figure S4

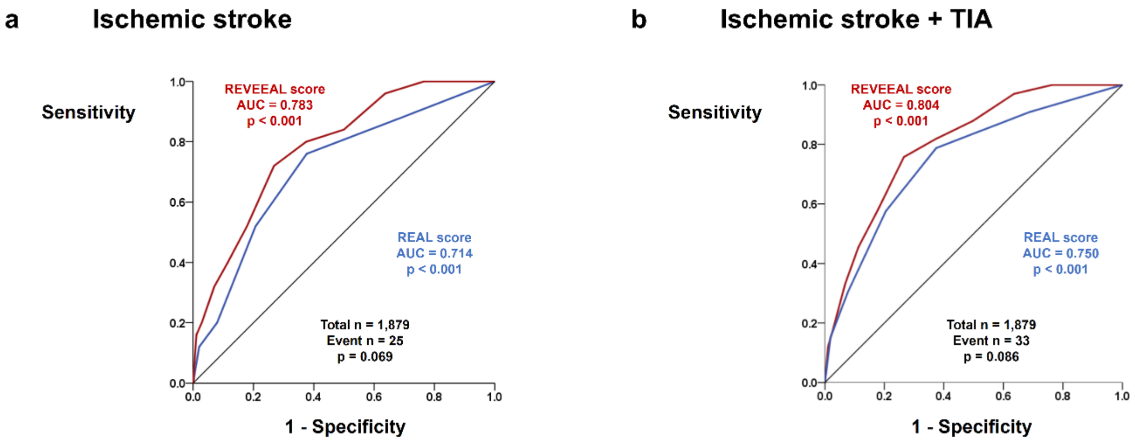

Supplementary Figure S4. Comparison of REVEEAL and REAL scoring system.

Statistical tendency for improved predictive value for ischemic stroke was observed when TTE and TEE findings were integrated to the scoring system.

AUC: area under curve; TIA: transient ischemic attack.
